# The Efficacy and Safety of Zhengqing Fengtongning for Knee Osteoarthritis: A Systematic Review and Meta-Analysis of Randomized Clinical Trials

**DOI:** 10.1155/2022/2768444

**Published:** 2022-01-20

**Authors:** Zeling Huang, Xiao Mao, Junming Chen, Junjun He, Shanni Shi, Miao Gui, Hongjian Gao, Zhenqiang Hong

**Affiliations:** ^1^Fujian University of Traditional Chinese Medicine, Fuzhou 350122, Fujian, China; ^2^Zhoupu Community Health Service Center of XiHu District of Hangzhou, Hangzhou 310024, Zhejiang, China; ^3^Key Laboratory of Orthopedics & Traumatology of Traditional Chinese Medicine and Rehabilitation Ministry of Education (Fujian University of TCM), Fuzhou 350122, Fujian, China

## Abstract

**Background:**

Zhengqing Fengtongning release tablet (ZQFTN) is a proprietary Chinese medicine preparation of sinomenine, the main active component of the traditional Chinese medicine (TCM) *Sinomenium acutum*. It is used in China as a complementary and alternative medicine (CAM) for knee osteoarthritis (KOA). The objective of this study was to evaluate the clinical efficacy and safety of ZQFTN in KOA treatment.

**Method:**

Randomized controlled trials of ZQFTN in KOA treatment were searched in PubMed, Cochrane Library, China National Knowledge Infrastructure, Chinese Scientific Journals Database, and Wanfang database. Two reviewers independently conducted the screening, extracted the data, and assessed the methodological quality. Statistical analysis was performed using RevMan 5.3 software.

**Results:**

Eighteen studies were assessed that included 1512 participants (757 in the treatment group and 755 in the control group). The results showed that compared with the control group, the Visual Analogue Scale (standardized mean difference (SMD) = −0.87, 95% confidence interval (CI): [−1.08, −0.66], *P* < 0.001), Western Ontario and Mc Master University (WOMAC) Osteoarthritis Index pain score (SMD = −0.67, 95% CI: [−0.88, −0.46], *P* < 0.001), WOMAC stiffness score (SMD = −0.53, 95% CI: [−0.86, −0.20], *P*=0.001), WOMAC function score (SMD = −0.76, 95% CI: [−0.97, −0.55], *P* < 0.001), serum interleukin-1*β* level (SMD = −4.36, 95% CI: [−6.41, −2.31], *P* < 0.001), and serum tumor necrosis factor-*α* level (SMD = −8.45, 95% CI: [−11.20, −5.69], *P* < 0.001) of the ZQFTN treatment group were lower, and the total effective rate was higher relative risk (*RR* = 1.15, 95% CI [1.07, 1.23], *P* < 0.001). There was no significant difference in the incidence of adverse reactions between the two groups (*RR* = 0.96, 95% CI: [0.69, 1.35], *P*=0.82).

**Conclusion:**

ZQFTN can effectively relieve knee pain, morning stiffness, and daily activity function disorders, reduce the expression of inflammatory factors in serum, and improve the total clinical response rate without increasing the incidence of adverse reactions. Therefore, ZQFTN has considerable potential as a CAM for KOA. However, due to the limitation of the quality of the included studies, the strength of this conclusion is affected. In the next step, multicenter, large sample, high-quality randomized controlled studies are needed to further confirm the present conclusion.

## 1. Introduction

Knee osteoarthritis (KOA) is a degenerative disease that occurs in the knee joint, with chronic joint pain, swelling, stiffness, and dysfunction as the main manifestations. With the increase in life expectancy and aging of the global population, its incidence is increasing and the burden on countries around the world is consequently becoming greater [1]. At present, the drugs used to treat KOA mainly include analgesics, intraarticular corticosteroids, nonsteroidal anti-inflammatory drugs (NSAIDs), and symptomatic slow acting drugs for osteoarthritis (SYSADOA) [2–4]. Although these drugs have certain effects on the pain and disease relief of osteoarthritis (OA) patients, they also increase the incidence of gastrointestinal ulcers and cardiovascular events, affecting their use by some patients [5]. Therefore, the need for a safe and effective option for OA treatment has transferred the focus of research from conventional drugs to complementary and alternative medicines (CAMs). Over time, ever increasing evidence has shown that traditional Chinese medicine (TCM) therapies, including acupuncture, galbanum oil, sesame oil, and Qigong, have favorable therapeutic potential as CAMs in OA treatment [6–9].

TCM has a long history, has the advantages of an accurate curative effect, safety and stability, and is a major research topic in the treatment of many difficult diseases [10]. Sinomenine (chemical structure: C19H23NO4, Mw 329.18) is a monomer alkaloid extracted from the TCM *Sinomenium acutum*, which has anti-inflammatory, analgesic, and immunomodulatory effects [11–14]. It can be used in the treatment of musculoskeletal diseases, neuropathy, cancer, and other diseases [15–19]. At present, Zhengqing Fengtongning release tablets (ZQFTN), sinomenine tablets, and sinomenine hydrochloride injection are used clinically in China [20]. To date, many clinical studies have been reported on the treatment of KOA with ZQFTN [21–38], but there remains a lack of relevant evidence-based medical studies on its efficacy and safety. Because there is no systematic review of oral ZQFTN for KOA treatment, whether ZQFTN can be used as a CAM for clinical KOA treatment remains inconclusive, which complicates the clinician's decision. Therefore, we conducted a meta-analysis on the efficacy and safety of ZQFTN in KOA treatment using evidence-based medicine for guidance.

## 2. Materials and Methods

The systematic review protocol was developed with guidance from the Preferred Reporting Items for Systematic Reviews and Meta-Analyses (PRISMA) statement and is registered in PROSPERO (CRD42021284282).

### 2.1. Search Strategy

Randomized controlled trials (RCTs) of ZQFTN in the treatment of KOA were searched in PubMed, Cochrane Library, China National Knowledge Infrastructure, Chinese Scientific Journals Database, and Wanfang database. The retrieval time is from a database construction to August 31, 2021. The retrieval strategy adopted the combination of subject words and free words. The key words were as follows: “Osteoarthritis” “knee osteoarthritis” “KOA” “Zhengqing Fengtongning release tablets” “Zhengqing Fengtongning” “Sinomenine,” and “Sinomenium”. The search strategy was as follows, taking PubMed as an example:

“Osteoarthritis” [Title/Abstract] OR “knee osteoarthritis” [Title/Abstract] OR “KOA” [Title/Abstract]
“Zhengqing Fengtongning release tablets” [Title/Abstract] OR “Zhengqing Fengtongning” [Title/Abstract] OR “Sinomenine” [Title/Abstract] OR “Sinomenium” [Title/Abstract]
“Randomized controlled trial” [Title/Abstract] OR “random trials” [Title/Abstract] OR “Controlled clinical trial” [Title/Abstract]
(1) and (2) and/or (3)

### 2.2. Inclusion and Exclusion Criteria

#### 2.2.1. Inclusion Criteria

The inclusion criteria were as follows: (1) Study type: RCTs, no language limitation. (2) Participants: patients should be clearly diagnosed with KOA. No restrictions on country, race, age, or gender. (3) Experimental group: in the treatment group, ZQFTN was taken orally alone or combined with other therapies. (4) Control group: any type of control group, including NSAIDs and SYSADOA among others. (5) Outcomes: total effective rate, Visual Analog Scale (VAS), Western Ontario and Mc Master University (WOMAC) Osteoarthritis Index, serum interleukin-1*β* (IL-1*β*) level, serum tumor necrosis factor-*α* (TNF-*α*) level, and adverse events.

#### 2.2.2. Exclusion Criteria

The exclusion criteria were as follows:

1. Repeated publications
2. Full-text literature is not available
3. Studies with incomplete data and information

### 2.3. Literature Screening and Data Extraction

Two reviewers (Zeling Huang and Xiao Mao) searched the literature and screened it independently according to the inclusion and exclusion criteria. We used standard data extraction methods to extract data. The basic information, sample characteristics, intervention measures, outcome, and other data, which were included in the article, were extracted by two reviewers (Zeling Huang and Xiao Mao). In the case of any inconsistency occurring in the result, this was further discussed by the two researchers or scrutinized by the third reviewer (Zhenqiang Hong).

### 2.4. Quality Assessment of the Included Studies

A bias risk assessment was conducted by two reviewers (Zeling Huang and Xiao Mao) based on the bias risk assessment tool recommended in the Cochrane manual [39, 40]. The details that were assessed were as follows: (1) random sequence generation; (2) allocation concealment; (3) blinding of participants and personnel; (4) blinding of outcome assessment; (5) incomplete outcome data; (6) selective reporting; and (7) others. Make high risk, low risk, or unclear judgments for each item. Any disagreements were resolved by the third reviewer (Zhenqiang Hong).

### 2.5. Statistical Analysis

Review Manager (RevMan) (Computer program), version 5.3 (the Nordic Cochrane Centre, the Cochrane Collaboration, Copenhagen, Denmark, 2014), was used to analyze the collected clinical research data. The enumeration data were evaluated using the relative risk (RR) and 95% confidence interval (CI), and the measurement data were combined using the standardized mean difference (SMD) and 95% CI. Analysis was performed using a fixed or random effects model according to the heterogeneity. The percentage of heterogeneity in the study was determined by the I^2^ statistic; if the *I*^2^ < 50%, the heterogeneity among the included studies was considered to be small and the fixed effect model was adopted. If *I*^2^ ≥ 50%, the heterogeneity among the included studies was considered significant, and the random effect model was adopted [41]. Subgroup analysis was conducted according to different treatments in the treatment group, and sensitivity analysis was also used to analyze the sources of heterogeneity. A value *P* < 0.10 was considered to suggest statistical heterogeneity and prompted random effects modeling.

## 3. Results

### 3.1. Literature Search Results

**Figure 1:**
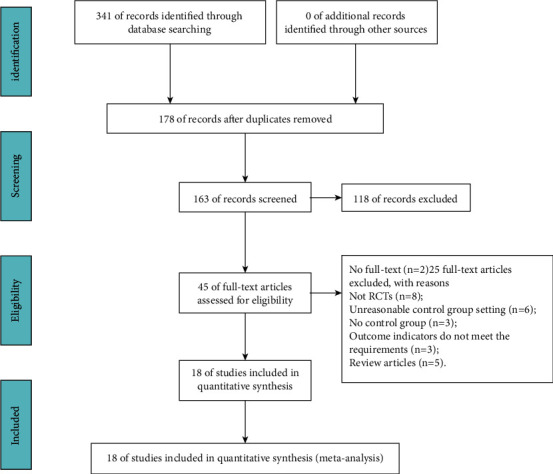
Process of searching and screening studies.

We initially retrieved 341 articles (Figure 1). We subsequently removed 178 duplicate articles manually, leaving 163 articles. Of these 163 articles, 118 were excluded after reading the title and abstract. Of the remaining articles, two lacked full text, and 25 were excluded because they failed to meet the inclusion criteria for complete reading. The present study eventually included 18 articles [21–38].

### 3.2. Characteristics of the Included Studies

The study of all included papers was a single-center, randomized controlled trial undertaken in China. In total, there were 757 cases in the experimental group and 755 cases in the control group. Except for three studies [28, 31, 33] that did not indicate drug sources, ZQFTN in the other fifteen studies was all produced by Hunan Zhengqing Pharmaceutical Group Co., Ltd. (Huaihua, Hunan, China). The included studies on the ZQFTN dosage are not uniform. Six studies [25, 26, 28, 34, 37, 38] defined ZQFTN alone as the experimental group, with a total of 209 patients, while the control group used SYSADOA, with a total of 209 patients. In seven studies [22, 23, 27, 31–33, 35], ZQFTN combined with SYSADOA was defined as the experimental group, with 318 patients in total, and SYSADOA was used by the control group, with 315 patients in total. In three studies [21, 24, 29], ZQFTN combined with NSAIDs was defined as the experimental group, with a total of 145 patients, and NSAIDs were used by the control group, with a total of 146 patients. In two studies [30, 36], 85 patients were treated with ZQFTN combined with sodium hyaluronate injection as the experimental group, and 85 patients were treated with sodium hyaluronate injection in the control group. The characteristics of the included studies are presented in Table 1.

### 3.3. Methodological Quality of Included Studies

**Figure 2:**
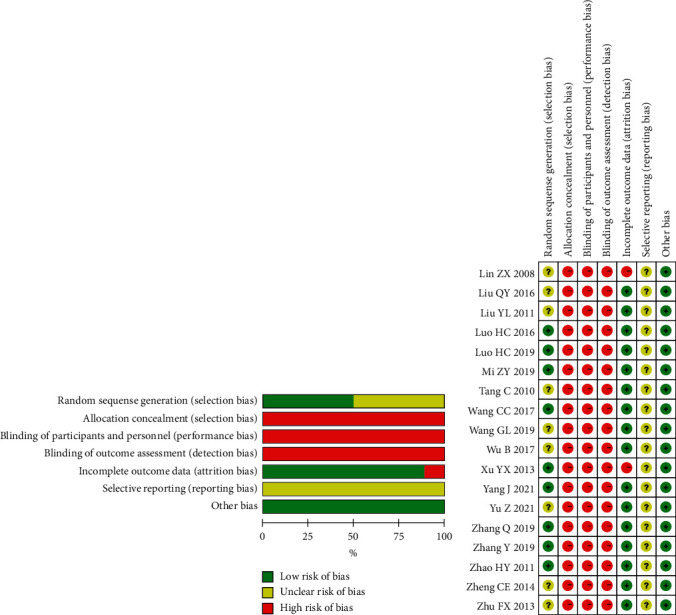
Risk of bias summary and risk of bias graph.

Most of the included studies were of low quality because of unclear randomization, inefficient allocation concealment, inadequate blinding, or described withdrawals and dropouts. Nine studies [24, 25, 30–33, 35–37] were grouped by the random number table method (low risk), and nine studies [21–23, 26–29, 34, 38] did not indicate a specific randomization method (unclear). Eighteen studies [21–38] did not implement allocation hiding (high risk) or blinding (high risk), while sixteen studies [23, 24, 26–38] had complete data (low risk). Because none of the eighteen studies had clinical trial registration in advance, the results of selective reporting were unclear, and none of the eighteen studies found other sources of bias (low risk). The risk of bias assessment is summarized in Figure 2.

### 3.4. Treatment Effects

#### 3.4.1. Total Effective Rate

**Figure 3:**
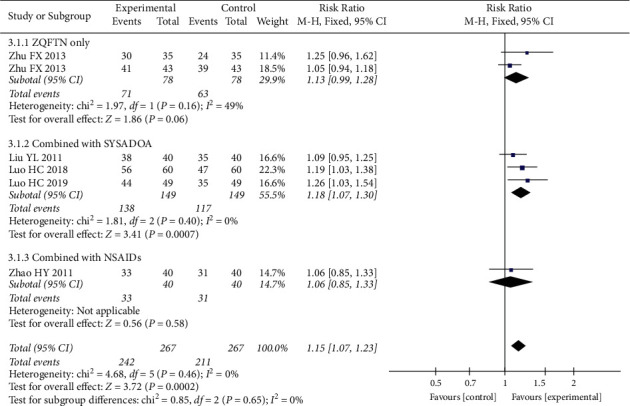
Forest plot of total effective rate.

The total effective rate is an important indicator for assessing the effect of treatment, which is mainly based on the changes in the patient's clinical symptoms before and after treatment. A total of seventeen of the included studies reported the total effective rate, but their criteria for judging the treatment effect included the WOMAC score, Lequesne index, and hospital for special surgery knee score. To enhance the strength of the results, we only performed meta-analysis on the six studies that used WOMAC scores to determine the total effective rate, involving a total of 534 patients, with 267 in the experimental group and 267 in the control group. The results of heterogeneity analysis showed good homogeneity among the included studies (*P*=0.46, *I*^2^ = 0%), and the fixed effect model was used for analysis. The results showed that the total effective rate of the experimental group was higher than that of the control group (*RR* = 1.15, 95% CI: [1.07, 1.23], *P* < 0.001). The analysis was divided into three subgroups according to different treatment methods of the experimental group. The results of the subgroup analysis showed that the total effective rate of ZQFTN alone was equivalent to that of SYSADOA alone (MD = 1.13, 95% CI: [0.99, 1.28], *P*=0.06). The total effective rate of ZQFTN combined with SYSADOA was higher than that of SYSADOA alone (RR = 1.18, 95% CI: [1.07, 1.30], *P* < 0.001). The total effective rate of ZQFTN combined with NSAIDs was equivalent to that of NSAIDs alone (RR = 1.06, 95% CI: [0.85, 1.33], *P*=0.03) (Figure 3).

#### 3.4.2. VAS

**Figure 4:**
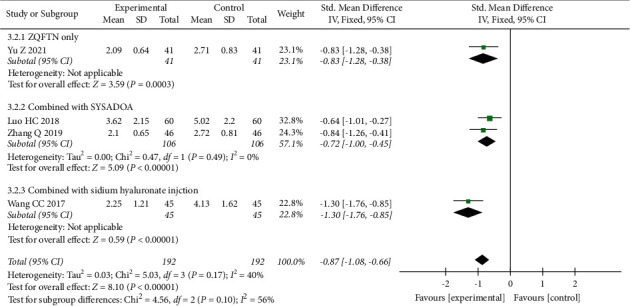
Forest plot of VAS.

Four studies [30, 31, 35, 38] reported a pain VAS after treatment, involving a total of 294 patients, 147 in the experimental group, and 147 in the control group. Heterogeneity analysis results showed good homogeneity among the included studies (*P*=0.17, *I*^2^ = 40%), and the fixed effect model was used for analysis. Results showed that the VAS of the experimental group was lower than that of the control group after treatment (SMD = −0.87, 95% CI: [−1.08, −0.66], *P* < 0.001). The results of subgroup analysis showed that the VAS of ZQFTN alone was lower than that of SYSADOA after treatment (SMD = −0.83, 95% CI: [−1.28, −0.38], *P* < 0.001). The VAS of the ZQFTN combined with the SYSADOA group was lower than that of SYSADOA alone after treatment (SMD = −0.72, 95% CI: [−1.00, −0.45], *P* < 0.001). The VAS of ZQFTN combined with sodium hyaluronate injection was lower than that of sodium hyaluronate injection alone after treatment (SMD = −1.30, 95% CI: [−1.76, −0.85], *P* < 0.001) (Figure 4).

#### 3.4.3. WOMAC Pain Score

**Figure 5:**
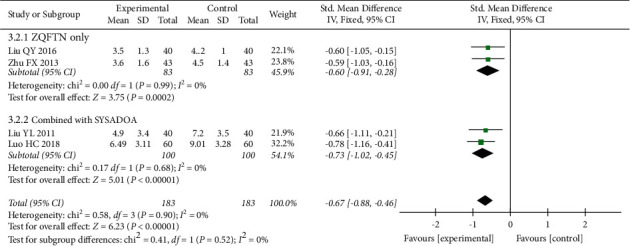
Forest plot of WOMAC pain score.

Four studies [23, 26, 28, 31] reported the WOMAC pain score after treatment, involving 366 patients (183 in the experimental group and 183 in the control group). Heterogeneity analysis showed good homogeneity among the included studies (*P*=0.90, *I*^2^ = 0%), and the fixed effect model was used for the analysis. The results showed that after treatment, the WOMAC pain score in the experimental group was lower than that in the control group (SMD = −0.67, 95% CI: [−0.88, −0.46], *P* < 0.001). The results of subgroup analysis showed that the WOMAC pain score of ZQFTN alone was lower than that of SYSADOA after treatment (SMD = −0.60, 95% CI: [−0.91, −0.28], *P* < 0.001). The WOMAC pain score of ZQFTN combined with SYSADOA was lower than that of SYSADOA alone after treatment (SMD = −0.73, 95% CI: [−1.02, −0.45], *P* < 0.001) (Figure 5).

#### 3.4.4. WOMAC Stiffness Score

**Figure 6:**
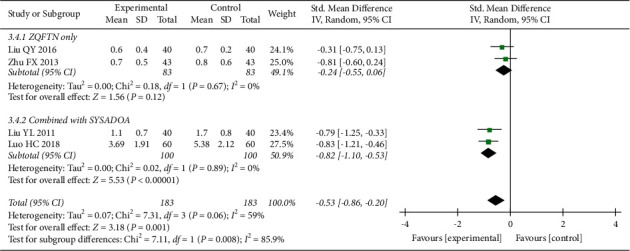
Forest plot of WOMAC stiffness score.

Four studies [23, 26, 28, 31] reported the WOMAC stiffness score after treatment, involving 366 patients (183 in the experimental group and 183 in the control group). The results of the heterogeneity analysis showed that there was significant heterogeneity among the included studies (*P*=0.06, *I*^2^ = 59%), and the random effect model was used to analyze the results. After treatment, the WOMAC stiffness score in the experimental group was lower than that in the control group (SMD = −0.53, 95% CI: [−0.86, −0.20], *P*=0.001). Subgroup analysis showed that there was no significant difference in the WOMAC stiffness score between the ZQFTN and SYSADOA groups after treatment (SMD = −0.24, 95% CI: [−0.55, −0.06], *P*=0.12). The WOMAC stiffness score of ZQFTN combined with SYSADOA was lower than that of SYSADOA alone after treatment (SMD = −0.82, 95% CI: [−1.10, −0.53], *P* < 0.001) (Figure 6).

#### 3.4.5. WOMAC Function Score

**Figure 7:**
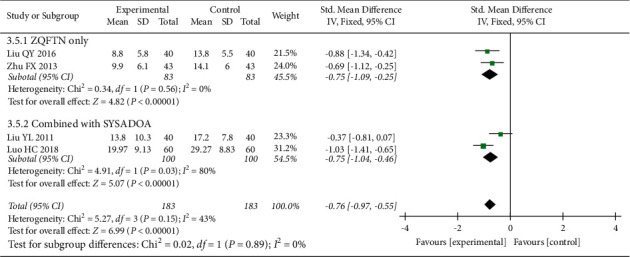
Forest plot of WOMAC function score.

Four studies [23, 26, 28, 31] reported the WOMAC function score after treatment, involving 366 patients (183 in the experimental group and 183 in the control group). Heterogeneity analysis showed good homogeneity among the included studies (*P*=0.15, *I*^2^ = 43%), and the fixed effect model was used for the analysis. The results showed that after treatment, the WOMAC function score in the experimental group was lower than that in the control group (SMD = −0.76, 95% CI: [−0.97, −0.55], *P* < 0.001). The results of the subgroup analysis showed that the WOMAC function score of ZQFTN alone was lower than that of SYSADOA after treatment (SMD = −0.78, 95% CI: [−1.09, −0.46], *P* < 0.001). The WOMAC function score of ZQFTN combined with SYSADOA was lower than that of SYSADOA alone after treatment (SMD = −0.75, 95% CI: [−1.04, −0.46], *P* < 0.001) (Figure 7).

### 3.5. Serum IL-1*β* Level

**Figure 8:**
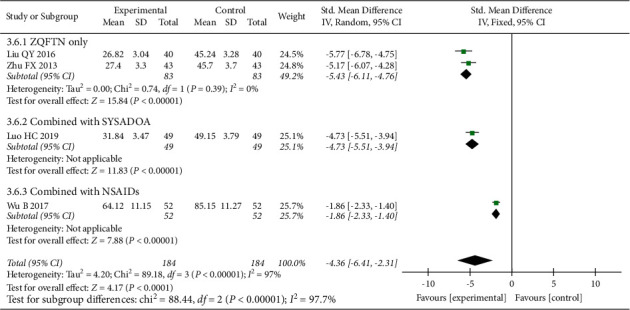
Forest plot of serum IL-1*β* level.

Four studies [15, 16, 24, 28] reported the serum IL-1*β* level after treatment, involving 368 patients (184 in the experimental group and 184 in the control group). The results of the heterogeneity analysis showed that there was significant heterogeneity among the included studies (*P* < 0.001, I^2^ = 97%), and the random effect model was used to analyze the results. The results showed that the serum IL-1*β* level of the experimental group was lower than that of the control group after treatment (SMD = −4.36, 95% CI: [−6.41, −2.31], *P* < 0.001). The results of subgroup analysis showed that the serum IL-1*β* level of ZQFTN alone was lower than that of SYSADOA after treatment (SMD = −5.43, 95% CI: [−6.11, −4.76], *P* < 0.001). The serum IL-1*β* level of the ZQFTN combined with SYSADOA group was lower than that of SYSADOA alone after treatment (SMD = −4.73, 95% CI: [−5.51, −3.94], *P* < 0.001). The serum IL-1*β* level of ZQFTN combined with NSAIDs was lower than that of NSAIDs alone after treatment (SMD = −1.86, 95% CI: [−2.33, −1.40], *P* < 0.001) (Figure 8).

### 3.6. Serum TNF-*α* Level

**Figure 9:**
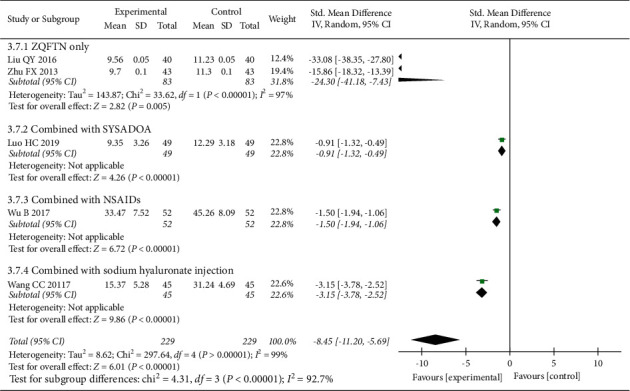
Forest plot of serum TNF-*α* level.

Five studies [15, 16, 24, 28, 30] reported the serum TNF-*α* level after treatment, involving 458 patients (229 in the experimental group and 229 in the control group). The results of the heterogeneity analysis showed that there was significant heterogeneity among the included studies (*P* < 0.001, *I*^2^ = 99%), and the random effect model was used to analyze the results. The results showed that the serum TNF-*α* level of the experimental group was lower than that of the control group after treatment (SMD = −8.45, 95% CI: [−11.20, −5.69], *P* < 0.001). The results of the subgroup analysis showed that the serum TNF-*α* level of ZQFTN alone was lower than that of SYSADOA after treatment (SMD = −24.30, 95% CI: [−41.18, −7.43], *P*=0.005). The serum TNF-*α* level of the ZQFTN combined with SYSADOA group was lower than that of SYSADOA alone after treatment (SMD = −0.91, 95% CI: [−1.32, −0.49], *P* < 0.001). The serum TNF-*α* level of ZQFTN combined with NSAIDs was lower than that of NSAIDs alone after treatment (SMD = −1.50, 95% CI: [−1.94, −1.06], *P* < 0.001). The serum TNF-*α* level of ZQFTN combined with sodium hyaluronate injection was lower than that of sodium hyaluronate injection alone after treatment (SMD = −3.15, 95% CI: [−3.78, −2.52], *P* < 0.001) (Figure 9).

### 3.7. Adverse Events

**Figure 10:**
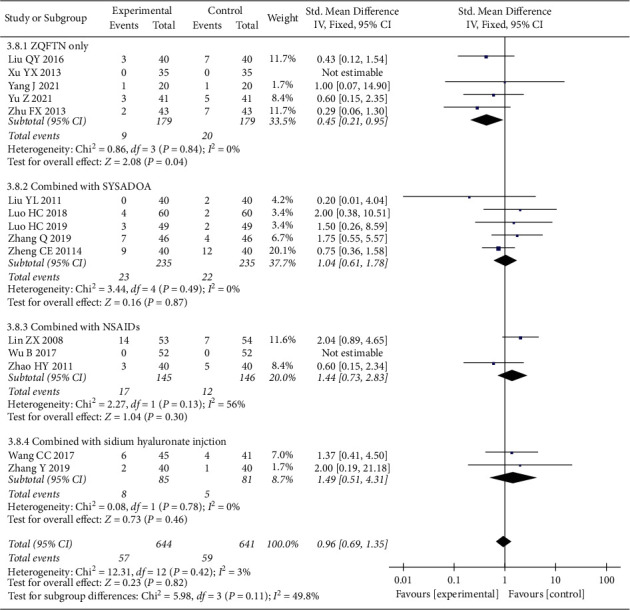
Forest plot of adverse events.

Fifteen studies [21, 23–32, 35–38] reported the adverse events after treatment, involving 1285 patients (644 in the experimental group and 641 in the control group). Heterogeneity analysis showed good homogeneity among the included studies (*P*=0.42, *I*^2^ = 3%), and the fixed effect model was used for the analysis. The results showed that there was no significant difference between the experimental group and the control group (*RR* = 0.96, 95% CI: [0.69, 1.35], *P*=0.82). The results of the subgroup analysis showed that ZQFTN alone had less adverse events than SYSADOA alone (*RR* = 0.45, 95% CI: [0.21, 0.95], *P*=0.04). There was no significant difference between the ZQFTN combined with SYSADOA group and the SYSADOA group (*RR* = 1.04, 95% CI: [0.61, 1.78], *P*=0.87). There was no significant difference in adverse events between ZQFTN combined with NSAIDs and NSAIDs alone (SMD = 1.44, 95% CI: [0.73, 2.83], *P*=0.30). There was no significant difference in adverse events between ZQFTN combined with sodium hyaluronate injection and sodium hyaluronate injection alone (*R*R = 1.49, 95% CI: [0.51, 4.31], *P*=0.46) (Figure 10).

### 3.8. Publication Bias

**Figure 11:**
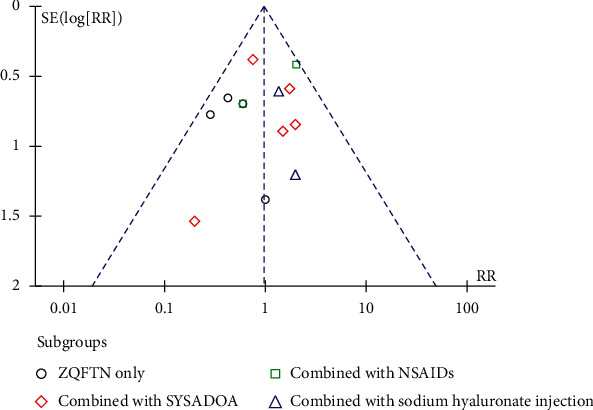
Publication bias of the funnel plot of adverse events.

The inverted funnel plot of publication bias was generated with the adverse reactions as indicators, and the scatter point distribution of each study was asymmetric, suggesting the possibility of publication bias in this study (Figure 11).

## 4. Discussion

*Sinomenium acutum* is the vine stem of the *Asteraceae* plant and Chinese *Anseraceae* plant among others. It is a TCM, which has the function of dispelling wind dampness, channelling channels and collaterals, and relieving urination, and is often used to treat rheumatoid arthritis, OA, and gout arthritis [42]. Sinomenine is an alkaloid extracted from *Sinomenium acutum* and is the main active ingredient of *Sinomenium acutum*. Experimental studies have found that sinomenine has clear anti-inflammatory and analgesic effects. Its anti-inflammatory effect mainly results from its selective inhibition of cyclooxygenase-2 activity, whereby capillary permeability is reduced by downregulating prostaglandin E synthesis, or preventing histamine-induced capillary permeability increase by inhibiting the release of various inflammatory mediators, thus blocking inflammatory infiltration and exudation. Sinomenine also has anticoagulation and antiembolism effects and reduces tissue damage [20]. Studies have shown that sinomenine can reduce synovial inflammation and cartilage degeneration by inhibiting the expression of inflammatory factors and chondrocyte apoptosis, thus delaying the progression of osteoarthritis [43]. ZQFTN as an oral preparation of sinomenine has been used ever more frequently in the clinical treatment of OA.

### 4.1. Effectiveness of ZQFTN in Treating KOA

Through a literature search, it was found that ZQFTN could be used as a complementary drug in combination with SYSADOA, NSAIDs, or sodium hyaluronate, or used alone as an alternative drug for KOA. The results showed that ZQFTN has good efficacy, but due to the influence of inconsistent treatment methods, insufficient sample size, and other factors, the conclusions were frequently unconvincing and the evidence basis was not strong. To determine the effectiveness of oral ZQFTN in KOA treatment, 18 RCTs were included in the present study, including 1512 KOA patients. The results of the meta-analysis showed that ZQFTN could effectively relieve knee pain, morning stiffness, and daily activity disturbance. The VAS and WOMAC scores were lower than the control group, and the total clinical effectiveness rate was higher than the control group, indicating that ZQFTN had significant clinical efficacy as a CAM for KOA. The results of serum IL-1*β* and TNF-*α* showed that their respective levels in the experimental group were lower than those in the control group, suggesting that the mechanism of ZQFTN in treating KOA may be associated with its ability to reduce inflammation.

### 4.2. Safety of ZQFTN in Treating KOA

Adverse events related to drug treatment were recorded in 15 studies, and meta-analysis results showed that there was no significant difference in the incidence of adverse reactions between the experimental and control groups. The reported adverse reactions were mainly gastrointestinal, including nausea and diarrhea, and allergic reactions, such as pruritus and rash, which were consistent with the adverse events recorded in the instructions of ZQFTN, SYSADOA, and NSAIDs. Eight studies monitored the changes of blood routine and liver and kidney functions during medication, including 737 KOA patients. The results showed that a total of five patients in the experimental groups displayed a slight increase in transaminases, while a further seven patients in control groups showed a slight increase in transaminases, which returned to normal after symptomatic treatment. These results indicated that the use of ZQFTN did not increase the risk for adverse events and that it was safe as a CAM for KOA.

### 4.3. Limitations of the Study

Through a comprehensive analysis of the included literature, the following problems were found to generally exist in this literature: (1) There was no allocation concealment or blind method in all studies, and strict and careful experimental design was lacking. (2) The sample size of some of the literature was small, and the calculation basis of sample size was not given. (3) There were differences in medication duration and dosage in the included studies, which were not conducive to the formation of standardized medication guidance. (4) Because there is no standardized ZQFTN in other countries, all analyzed studies were performed in China, which may have led to a certain bias.

## 5. Conclusion

This study demonstrated that ZQFTN has high clinical efficacy and safety in the treatment of KOA and thus has considerable potential as a CAM for KOA. However, due to the limitation of the quality of included studies, the strength of this conclusion is affected. In the next step, multicenter, large sample, high-quality randomized controlled studies are needed to further confirm the present conclusion.

## Figures and Tables

**Table 1:** Characteristics of the included studies.

Author, year	Sample size		Sex (male/female)		Age (years)		Treatment		Treatment cycle	Outcomes
	EG	CG	EG	CG	EG	CG	EG	CG		
Lin ZX, 2008 [21]	53	54	15/38	16/38	63.5 ± 8.7	65.7 ± 9.2	ZQFTN 120 mg bid + CG	Diclofenac sodium sustained-release tablets	6 months	TER, AE
Tang C, 2010 [22]	33	30	18/15	16/14	58.5 ± 8.3	59.8 ± 8.1	ZQFTN 60 mg bid + CG	Glucosamine sulfate capsules	6 weeks	TER
Liu YL, 2011 [23]	40	40	19/21	22/18	63 ± 8	62 ± 8	ZQFTN 60 mg tid + CG	Glucosamine hydrochloride capsules	3 months	TER, WOMAC, AE
Zhao HY, 2011 [24]	40	40	-	-	59.975 ± 11.077	61.200 ± 10.649	ZQFTN 120 mg bid + CG	Celecoxib	8 weeks	TER, AE
Xu YX, 2013 [25]	35	35	18/17	17/18	52	53	ZQFTN 60 mg bid	Diacerein capsules	8 weeks	TER, AE
Zhu FX, 2013 [26]	43	43	12//31	13/30	65.17 ± 8.73	64.93 ± 9.12	ZQFTN 60 mg bid	Glucosamine hydrochloride capsules	12 weeks	TER, WOMAC, IL-1*β*, TNF-*α*, AE
Zheng CE, 2014 [27]	40	40	11//29	9/31	60.8 ± 6.6	61.2 ± 5.8	ZQFTN 60 mg bid + CG	Glucosamine sulfate capsules	1 months	TER, AE
Liu QY, 2016 [28]	40	40	18/22	15/25	68.15 ± 7.8	65.28 ± 1.20	ZQFTN 60 mg bid	Glucosamine hydrochloride capsules	10 weeks	TER, WOMAC, IL-1*β*, TNF-*α*, AE
Wu B, 2017 [29]	52	52	36/16	34/18	62.19 ± 7.20	62.24 ± 7.15	ZQFTN 20 mg tid + CG	Meloxicam	4 weeks	TER, IL-1*β*, TNF-*α*, AE
Wang CC, 2017 [30]	45	45	16/29	18/27	49.59 ± 5.70	51.63 ± 5.12	ZQFTN 120 mg bid + CG	Sodium hyaluronate injection	10 weeks	VAS, TNF-*α*, AE
Luo HC, 2018 [31]	60	60	21/39	25/35	61.28 ± 10.12	59.97 ± 11.03	ZQFTN 60 mg bid + CG	Diacerein capsules	8 weeks	TER, VAS, WOMAC, AE
Luo HC, 2019 [32]	49	49	13/36	14/35	57.49 ± 10.52	59.92 ± 10.89	ZQFTN 60 mg bid + CG	Glucosamine hydrochloride capsules	12 weeks	TER, IL-1*β*, TNF-*α*, AE
Mi ZY, 2019 [33]	50	50	22/28	19/31	62.7 ± 5.3	60.9 ± 5.0	ZQFTN 60 mg bid + CG	Diacerein capsules	2 months	TER
Wang GL, 2019 [34]	30	30	16/14	17/13	32.2 ± 8.4	40.5 ± 8.2	ZQFTN 60 mg bid	Diacerein capsules	8 weeks	TER
Zhang *Q*, 2019 [35]	46	46	18/28	16/30	58.91 ± 5.63	58.72 ± 5.81	ZQFTN 120 mg bid + CG	Diacerein capsules	8 weeks	TER, VAS, AE
Zhang Y, 2019 [36]	40	40	22/18	21/19	52.6 ± 2.5	52.7 ± 2.4	ZQFTN 60 mg bid + CG	Sodium hyaluronate injection	5 weeks	TER, AE
Yang J, 2021 [37]	20	20	14//6	13//7	62.45 ± 4.25	62.50 ± 4.00	ZQFTN 60 mg tid	Glucosamine hydrochloride capsules	10 weeks	TER, AE
Yu Z, 2021 [38]	41	41	23/18	24/17	60.78 ± 8.51	61.42 ± 8.23	ZQFTN 120 mg bid	Diacerein capsules	3 months	TER, VAS, AE

EG: experimental group; CG: control group; -: not mentioned; ZQFTN: Zhengqing Fengtongning release tablets; TER: total effective rate; VAS: Visual Analog Scale; WOMAC: Western Ontario and Mc Master University Osteoarthritis Index; IL-1*β*: serum IL-1*β* level; TNF-*α*: serum TNF-*α* level; AE: adverse events.

## Data Availability

The table data used to support the findings of this study are included within the article. The figure data used to support the findings of this study are included within the figure files.

## References

[1] Safiri S., Kolahi A. A., Smith E. (2017). Global, regional and national burden of osteoarthritis 1990-2017: a systematic analysis of the Global Burden of Disease Study 2017. *Annals of the Rheumatic Diseases*.

[2] Katz J. N., Arant K. R., Loeser R. F. (2021). Diagnosis and treatment of hip and knee osteoarthritis. *JAMA*.

[3] Persson M. S. M., Stocks J., Varadi G. (2020). Predicting response to topical non-steroidal anti-inflammatory drugs in osteoarthritis: an individual patient data meta-analysis of randomized controlled trials. *Rheumatology*.

[4] Magni A., Agostoni P., Bonezzi C. (2021). Management of osteoarthritis: expert opinion on NSAIDs. *Pain and Therapy*.

[5] Pelletier M., Barr A. J., Cicuttini F. M. (2017). Osteoarthritis. *Nature Reviews Disease Primers*.

[6] Tu J. F., Yang J. W., Shi G. X. (2021). Efficacy of intensive acupuncture versus sham acupuncture in knee osteoarthritis: a randomized controlled trial. *Arthritis & Rheumatology*.

[7] Emami Razavi Z., Karimi M., Khamessi M. (2016). Effects of galbanum oil on patients with knee osteoarthritis: a randomized controlled clinical trial. *Traditional and Integrative Medicine*.

[8] Askari A., Ravansalar S. A., Naghizadeh M. M. (2019). The efficacy of topical sesame oil in patients with knee osteoarthritis: a randomized double-blinded active-controlled non-inferiority clinical trial. *Complementary therapies in medicine*.

[9] Leung K. C. W. (2021). Mind-body health benefits of traditional chinese qigong on women: a systematic review of randomized controlled trials. *Evidence-based Complementary and Alternative Medicine*.

[10] Wang L., Zhang X. F., Zhang X. (2020). Evaluation of the therapeutic effect of traditional Chinese medicine on osteoarthritis: a systematic review and meta-analysis. *Pain Research and Management*.

[11] Yamasaki H. (1976). Pharmacology of sinomenine, an anti-rheumatic alkaloid from sinomenium.

[12] Jiang W., Fan W., Gao T. (2020). Analgesic Mechanism of Sinomenine against Chronic Pain. *Pain Res Manag*.

[13] Zhou H., Liu J.-X., Luo J.-F. (2017). Suppressing mPGES-1 expression by sinomenine ameliorates inflammation and arthritis. *Biochemical Pharmacology*.

[14] Gao T., Hao J., Wiesenfeld-Hallin Z., Wang D. Q., Xu X. J (2013). Analgesic effect of sinomenine in rodents after inflammation and nerve injury. *European Journal of Pharmacology*.

[15] Huang R. Y., Pan H. d., Wu J.-q. (2019). Comparison of combination therapy with methotrexate and sinomenine or leflunomide for active rheumatoid arthritis: a randomized controlled clinical trial. *Phytomedicine*.

[16] Gao Z., Lin Y., Zhang P. (2019). Sinomenine ameliorates intervertebral disc degeneration via inhibition of apoptosis and autophagy in vitro and in vivo. *American Journal of Tourism Research*.

[17] Wang X., Liu Y., Zhang H. (2021). Sinomenine alleviates dorsal root ganglia inflammation to inhibit neuropathic pain via the p38 MAPK/CREB signalling pathway. *European Journal of Pharmacology*.

[18] Song L., Zhang H., Hu M. (2020). Sinomenine inhibits hypoxia induced breast cancer side population cells metastasis by PI3K/Akt/mTOR pathway. *Bioorganic & Medicinal Chemistry*.

[19] Xu F., Li Q., Wang Z., Cao X. (2019). Sinomenine inhibits proliferation, migration, invasion and promotes apoptosis of prostate cancer cells by regulation of miR-23a. *Biomedicine & pharmacotherapy = Biomedecine & pharmacotherapie*.

[20] Wang X., Zhang Z. Y., Chou P. (2021). Research progress of Sinomenium acutum and Sinomenine and their related preparations. *Chinese Pharmaceutical Journal*.

[21] Lin Z. X., Cui Z. J., Dai Y. C. (2008). Clinical observation on 58 cases of knee osteoarthritis treated with Zhengqing Fengtongning combined with Western medicine. *Guiding Journal of TCM and Pharmacy*.

[22] Tang C., Cai X. Y., Lin X. J. (2010). Clinical observation of glucosamine combined with Zhengqing Fengtongning release tablets in the treatment of knee osteoarthritis. *China Modern Doctor*.

[23] Liu Y. L., Liu Y. M., Liu Y. X. (2011). Clinical observation of Zhengqing Fengtongning combined with Puli in the treatment of knee osteoarthritis. *Hebei Medical Journal*.

[24] Zhao H. Y., Huang Y. Z., Zhang J. (2011). 40 cases of knee osteoarthritis treated by Zhengqing Fengtongning combined with celecoxib. *Herald of Medicine*.

[25] Xv Y. X. (2013). *Clinical Study of Zhengqing Fengtongning Release Tablets in the Treatment of Osteoarthritis of the Knee*.

[26] Zhu F. X., Zhou R. H., Shi Y. H. (2013). Clinical study of Zhengqing Fengtongning in the treatment of knee osteoarthritis and its effect on cytokines. *Contemporary Medicine*.

[27] Zheng C. E., Liu Y. C., Zhang F. (2014). Clinical study of glucosamine sulfate combined with Zhengqing Fengtongning in the treatment of knee osteoarthritis. *Research of Integrated Traditional Chinese and Western Medicine*.

[28] Liu Q. Y. (2016). Clinical effect of Zhengqing Fengtongning on knee osteoarthritis and its effect on cytokiness. *Chronic Pathematology Journal*.

[29] Wu B., Zhong Q. H., Xun D. (2017). Efficacy of loxicam combined with Zhengqing Fengtongning in the treatment of patients with osteoarthritis and its effect on serum inflammatory indexes. *Heilongjiang Medicine Journal*.

[30] Wang C. C., Li M. Z., Gao Z. M. (2017). Analysis of the short-term efficacy of Zhengqing Fengtongning combined with sodium hyaluronate injection in the treatment of knee arthritis. *Pharmacology and Clinics of Chinese Materia Medica*.

[31] Luo H. C., Ou D. M. (2018). Clinical effect of sinomenine combined with diacetacin in the treatment of knee osteoarthritis. *Chinese Traditional Patent Medicine*.

[32] Luo H. C., Hu D. H. (2019). Efficacy and safety of glucosamine hydrochloride combined with sinomenine in the treatment of knee osteoarthritis. *Chinese Traditional Patent Medicine*.

[33] Mi Z. Y. (2019). Clinical effect analysis of Zhengqing Fengtongning release tablets combined with Shuangqiurein Capsule on patients with knee osteoarthritis. *Chinese Community Doctors*.

[34] Wang G. L. (2019). Clinical observation of Zhengqing Fengtongning release tablet in the treatment of knee osteoarthritis. *Medical Diet and Health*.

[35] Zhang Q., Chen W. Y. (2019). Observation on the curative effect of Zhengqing Fengtongning release tablets combined with diacerein in the treatment of knee osteoarthritis. *Drugs & Clinic*.

[36] Zhang Y., Liu F. R., Gu C. H. (2019). A randomized parallel control study of Zhengqing Fengtongning combined with sodium hyaluronate in the treatment of knee osteoarthritis. *Journal of Practical Traditional Chinese Internal Medicine*.

[37] Yang J. (2021). Clinical effect of Zhengqing Fengtongning on degenerative knee arthritis. *Inner Mongolia Journal of TCM*.

[38] Yv Z., Xv Y., Zang Y. S. (2021). Application of Zhengqing Fengtongning release tablets in the treatment of 41 cases of knee osteoarthritis. *World Latest Medicine Information*.

[39] Hopp L. (2015). Risk of bias reporting in cochrane systematic reviews. *International Journal of Nursing Practice*.

[40] Li J. L., Rong S., Zhou Z. (2021). The efficacy and safety of acupuncture for treating osteoporotic vertebral compression fracture- (OVCF-) induced pain: a systematic review and meta-analysis of randomized clinical trials. *Evid Based Complement Alternat Med*.

[41] Cordero C. P., Dans A. L. (2021). Key concepts in clinical epidemiology: detecting and dealing with heterogeneity in meta-analyses. *Journal of Clinical Epidemiology*.

[42] Hui W. Q., Wang X. X., Wang J. (2019). Research progress on natural components, clinical application and adverse reactions of Sinomenium acutum. *Journal of Modern Medicine & Health*.

[43] Zheng J., Wang R. H., Kou S. D. (2016). Effects of sinomenine on expression of vascular endothelial growth factor and nerve growth factor in cartilage and synovial membrane of rabbit knee osteoarthritis model. *Chinese Journal of Information on TCM*.

